# Clinical and Technological Evaluation of the Remineralising Effect of Biomimetic Hydroxyapatite in a Population Aged 6 to 18 Years: A Randomized Clinical Trial

**DOI:** 10.3390/bioengineering12020152

**Published:** 2025-02-05

**Authors:** Andrea Scribante, Saverio Cosola, Maurizio Pascadopoli, Annamaria Genovesi, Rebecca Andrea Battisti, Andrea Butera

**Affiliations:** 1 Unit of Orthodontics and Pediatric Dentistry, Section of Dentistry, Department of Clinical, Surgical, Diagnostic and Paediatric Sciences, University of Pavia, 27100 Pavia, Italy; 2 Unit of Dental Hygiene, Section of Dentistry, Department of Clinical, Surgical, Diagnostic and Paediatric Sciences, University of Pavia, 27100 Pavia, Italy; 3 Department of Stomatology, Tuscan Stomatologic Institute, Foundation for Dental Clinic, Research and Continuing Education, 55041 Camaiore, Italy; 4 Department of Dentistry, Unicamillus—Saint Camillus International University of Health and Medical Sciences, 00100 Rome, Italy

**Keywords:** remineralisation, biomimetic hydroxyapatite, pediatric patients, DIAGNOcam, DIAGNOdent Pen

## Abstract

The aim of this randomized clinical trial was to evaluate the efficacy of two different remineralising toothpastes in preventing dental caries and promoting oral health. Patients aged 6–18 years old with healthy and fully erupted first permanent molars (C1 and C2 DIAGNOdent scores) were enrolled and randomized into two groups according to the home-hydroxyapatite-based remineralising treatment used: the Trial group used zinc carbonate hydroxyapatite-based treatment (Biorepair Total Protective Repair), while the Control group used magnesium strontium carbonate hydroxyapatite conjugated with chitosan toothpaste (Curasept Biosmalto Caries Abrasion & Erosion). Dental and periodontal parameters were measured over a six-month period, including the DIAGNOdent Pen Index (primary outcome), BEWE Index, Plaque Index, Bleeding Score, Schiff Air Index, and ICDAS assessed with DIAGNOcam. A total of 40 patients were equally allocated in the two groups and finally analyzed. A significant reduction in the DIAGNOdent Pen score was reported in the Trial group after 1 month of treatment, while in the Control group, no significant change was found. The Trial group also showed a significant reduction in plaque levels after 3 months of treatment, while in the Control group, it occurred after 1 month. However, the Bleeding Score and Schiff Air Index showed no significant differences between the groups, suggesting that additional measures may be required to address gingival inflammation and hypersensitivity. The ICDAS index also showed no statistically significant changes, due to the limited duration of this study. Overall, zinc-hydroxyapatite-based toothpaste was more effective than magnesium strontium carbonate hydroxyapatite toothpaste in enhancing enamel remineralisation in the short-term period. The assigned treatments did not result in significant improvements in the oral indexes assessed in this study.

## 1. Introduction

Dental enamel is an acellular, highly mineralised tissue that cannot repair itself. Developmental defects or environmental influences can alter the opacity and colour of enamel, highlighting the critical impact of developmental insults on enamel integrity [1]. Over time, enamel is continuously exposed to external agents that can lead to mineral and tissue loss. The gradual demineralisation process occurs as enamel permeability decreases with the uptake of calcium, phosphate, and fluoride ions present in the oral cavity [2].

Dental caries is a multifactorial disease that progressively destroys the hard tissues of the tooth and is characterized by bacterial infection, proliferation, and demineralization [3]. Although socio-economic and behavioural factors also play an important role in the etiology of carious lesions, the development of this disease depends on the interplay of three main factors: acidogenic and acidophilic microorganisms, dietary carbohydrates, and host factors [4]. It results from an ecological imbalance in the oral microbiome, where acid-producing bacteria such as *Streptococcus mutans*, *non-mutans streptococci*, *Actinomyces*, and *Lactobacillus* lower the pH, leading to the demineralisation of hydroxyapatite crystals and proteolytic degradation of dental tissues [5].

Enamel demineralisation marks the initial stage of dental caries and manifests as a softened surface area that progresses to a sub-surface lesion. Clinically, it may be visible as a white spot and can occur within four weeks of prolonged acidic conditions. Continued exposure to acids produced by oral bacteria leads to a net loss of minerals, resulting in irreversible cavitation of the demineralised area [6]. Enamel becomes rough and porous when exposed to acids, making it susceptible to progression of the demineralisation process [7]. Enamel demineralisation and remineralisation are dynamic physicochemical processes regulated by fluctuations in pH. During demineralisation, acids dissolve enamel minerals, while remineralisation restores lost minerals when the oral environment becomes supersaturated with calcium and phosphate ions [5]. Saliva plays an important buffering role by neutralizing acids and promoting remineralisation. Advanced technologies have been developed to harness the natural remineralisation potential of saliva, making it a key focus for therapeutic intervention [7,8,9]. The process of remineralisation can be enhanced using fluoride and biomimetic materials [10,11]. These technologies work by improving the intrinsic ability of saliva to replenish lost minerals, thereby partially restoring enamel integrity [12]. Clinicians are increasingly considering remineralisation as a therapeutic strategy to counteract or prevent early carious lesions [13,14]. Various treatments could be tested in pediatric patients to improve hard and soft tissue health, including ozonized compounds [15], probiotics [16,17,18], and remineralising home toothpastes [19,20], but the additional evaluation of home products to be used daily would be desirable as an alternative to in-office procedures. Considering that modern technologies involving laser fluorescence and fibre-optic transillumination can provide a numerical and visual examination of the degree of demineralized hard tissues, DIAGNOdent Pen and DIAGNOcam [21] can be considered to numerically evaluate the effect of remineralising treatments in fissures of permanent primary molars in pediatric patients. Currently, among the available remineralising products, biomimetic hydroxyapatite has been widely investigated, for the fact that it is the constituent of enamel, dentin, and bone; therefore, it deposits on hard tissues, depositing and forming a new coating [13]; its incorporation into oral hygiene products has been proven to be successful, and it is widely acknowledged as fluoride [13,20,22].

Therefore, in order to provide proper oral care for dental patients in terms of enamel prevention and non-invasive domiciliary treatment, the present study aimed to compare the efficacy of two different biomimetic hydroxyapatite-based toothpastes in a 6-month study using DIAGNOdent Pen scores to evaluate the degree of demineralization of permanent first molars. DIAGNOcam was used to take photographs of the enrolled molars. Periodontal assessment was conducted by assessing basic erosive wear examination (BEWE), the Schiff Air Index (SAI), the Plaque Index (PI), and bleeding on probing (BoP).

The null hypothesis was that there would be no significant difference in the study variables between the two groups over time.

## 2. Materials and Methods

### 2.1. Trial Design

This study was a single-centre, parallel, active-controlled, randomized trial with an equal number of patients allocated to the two study groups. The Unit Internal Review Board approved the trial (ID: 2024-0313) and the trial was registered on clinicaltrials.gov (accessed on 10 January 2025) (NCT n°: NCT06425536).

### 2.2. Participants

This study was conducted at the Unit of Dental Hygiene, Section of Dentistry, Department of Clinical, Surgical, Diagnostic and Pediatric Sciences, University of Pavia, Italy. The study lasted 7 months; it started on the 1st of May 2024 and ended on the 30th of November 2024. Written informed consent was obtained from the parents of the patients to participate in the study. Patients were selected from children under routinary care. The following inclusion criteria were adopted for the enrolment of pediatric patients:

- Aged between 6 and 18 years.
- Permanent first molars erupted and completely healthy.
- Patients presenting C1 values (0–12) and C2 values (13–24) of the DIAGNOdent Pen of first molars.

The following exclusion criteria were adopted:

- Patients with DIAGNOdent Pen score on first molars > 25.
- Patients with groove sealings of sealed permanent first molars or composite restorations.
- Permanent first molars with extensive demineralisations (Molar Incisor Hypomineralization, fluorosis, white/brown spots).

### 2.3. Interventions and Outcomes

At baseline (T0), patients underwent an oral examination by an experienced calibrated operator (R.A.B.). The following indices were recorded to assess periodontal health: Schiff Air Index (SAI) [23]; Plaque Index (PI) and Bleeding on Probing (BoP) [24]; and basic erosive wear examination (BEWE) [25]. For hard tissue evaluation, DIAGNOdent Pen (2190 model, KaVo, Bieberach, Germany) and DIAGNOcam (DIAGNOcam Vision Full HD, KaVo) were used on all the primary first molars by the same operator [21]. DIAGNOdent Pen score (DP), a numerical score according to the degree of demineralization (higher values correspond to a higher degree of demineralization), was recorded; DIAGNOcam score (DC) was a visual calculation of the ICDAS [19] score through photographs recorded by DIAGNOcam itself.

Subsequently, nonsurgical mechanical periodontal debridement was performed using a piezoelectric instrument (Multipiezo, Mectron S.p.a., Carasco, Italy) and glycine powder with a specific handpiece (Mectron S.p.a., Carasco, Italy). Patients and parents were motivated and instructed in proper oral hygiene home care with tools specific to each patient. At the end of the oral hygiene session and motivation, the patients were randomly assigned by another operator (A.B.) to a group to receive the final instructions to use the toothpaste twice a day for 2 min for the entire duration of this study, as follows:

- Trial group: Biorepair Total Protective toothpaste containing zinc carbonate-substituted hydroxyapatite crystals [20].
- Control group: Curasept Biosmalto Caries & Erosion toothpaste containing fluorohydroxyapatite and magnesium strontium carbonate hydroxyapatite conjugated with chitosan [13]. This toothpaste was considered the control group because it is a fluoro-based formula and it is considered the gold standard for remineralization [13,25].

The compositions of the two toothpastes are shown in Table 1.

The time point recalls in which the oral parameters were re-evaluated were as follows: 1 month (T1), after 3 months (T2), and after 6 months (T3) when another mechanical debridement was performed.

### 2.4. Sample Size

The sample size was calculated considering alpha = 0.05 and power = 80% for two independent study groups. DIAGNOdent Pen score was considered the primary continuous outcome; therefore, the expected mean was 9.56 and the expected difference between the means was supposed to be 1.65 with a standard deviation of 3.73 [26]; the definite number of patients for this study was therefore 40, equally divided into the two study groups.

### 2.5. Randomisation and Blinding

The data analyst used a block randomization table for the generation of the random sequence considering a permuted block of 40 patients. Research Randomizer (version 4.0) was used by the data analyst (A.S.). The allocation was performed with sequentially numbered, opaque, sealed envelopes; afterwards, the calibrated operator (R.A.B.) performed the mechanical periodontal debridement, while the care provider assigned patients to the Trial group or Control group (A.B.). Patients, operators, and data analysts were blinded for allocation. With regard to the home protocol, the products were concealed [10].

### 2.6. Statistical Methods

R Software (R version 3.1.3, R Development Core Team, R Foundation for Statistical Computing, Wien, Austria) was used to perform statistical analysis. The mean and standard deviation were calculated for all the variables as descriptive statistics. The data normality of distributions was assessed with the Kolmogorov–Smirnov test. As data were not normally distributed, Kruskal–Wallis followed by Dunn’s multiple comparisons test were performed. A significance threshold of *p* < 0.05 was set for all the statistical tests.

## 3. Results

### 3.1. Participant Flow and Baseline Data

**Figure 1:**
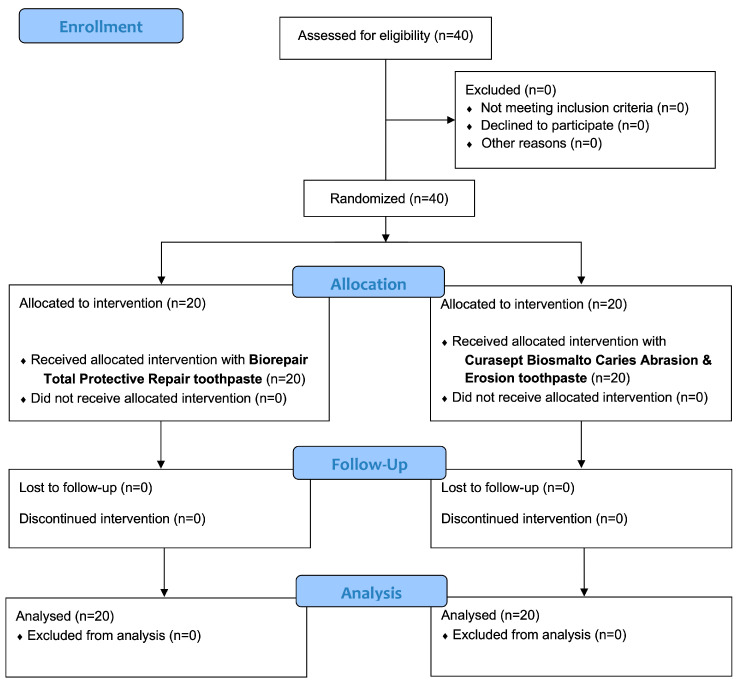
Description of the phases of this study according to CONSORT flow chart 2010.

A total of 40 patients were enrolled and allocated into two groups. No drop-outs were reported and all the patients concluded the follow-up of 6 months. Figure 1 shows the entire flow chart of this study. At the beginning of the study, patients exhibited a mean age of 13.3 ± 3.1 years (20 males and 20 females). For the Trial group, the mean age was 13.3 ± 3.1 years (8 females and 12 males), while for the Control group, it was 12.9 ± 3.1 years (12 males and 8 females).

### 3.2. Study Variables

The descriptive and inferential statistics of DP and DC scores are shown in Table 2.

For the DP score, the Trial group showed a statistically significant reduction from T0 to T1 (*p* < 0.05), with no other significant intragroup differences (*p* > 0.05). In the Control group, no significant differences were found (*p* < 0.05), and the same occurred for intergroup comparisons (*p* > 0.05).

**Figure 2:**
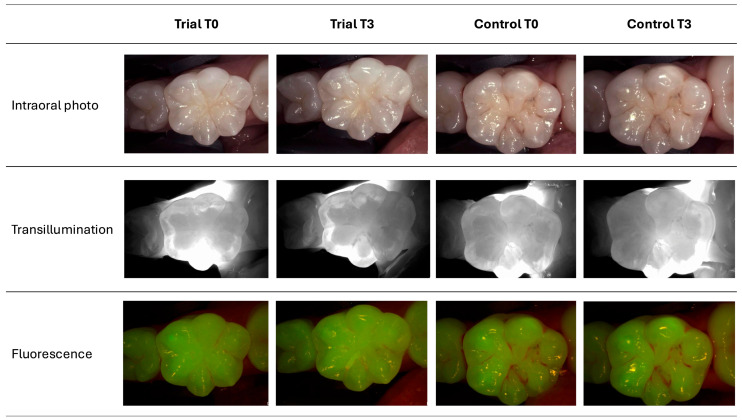
DIAGNOdent photographs of two experimental treatments at T0 and T3 time frames.

For the DC score, no significant inter- and intragroup differences were found (*p* > 0.05). In Figure 2, examples of photographs taken with DIAGNOcam from the Trial and Control groups before and after treatment are shown.

Regarding the oral indexes (Table 3), SAI, BoP, and BEWE did not show statistically significant inter- and intragroup differences (*p* > 0.05), although a gradual decrease was found in all the variables. Regarding PI, in the Trial group, a significant decrease was found in the T0–T2 comparison (*p* < 0.05), while in the Control group in the T0–T1 comparison (*p* < 0.05), no significant intergroup differences were found (*p* > 0.05).

## 4. Discussion

The health and economic burden of dental caries is considerable. The Global Burden of Diseases study published in 2019 [27] revealed that carious lesions in permanent teeth were the most prevalent and second most common of the 328 diseases studied, affecting 2.44 billion people globally. Despite advances in oral care prevention and treatment, dental caries remains highly prevalent and a challenge for clinicians and society, especially in developing countries [28]. The most common diagnostic tools used to detect caries include clinical examination and radiography. However, these methods have limitations as monitoring caries progression by visual examination is complex due to low reproducibility [29,30], while radiographs may underestimate the depth of carious lesions, have low sensitivity, are not suitable for initial caries, and expose patients to X-rays [31]. Among the modern technologies developed and currently available, DIAGNOdent Pen based on laser fluorescence provides simple and effective results by detecting differences in fluorescence between healthy and demineralised dental tissues [32]. Moreover, the DIAGNOcam camera, based on laser-induced fluorescence and transillumination, is another instrument capable of taking high-quality photographs of dental occlusal surfaces, allowing a more accurate enamel visual inspection useful for caries diagnosis [33].

In the present study, two remineralising toothpastes were tested in pediatric patients to evaluate the improvements in enamel remineralization and oral and periodontal condition. In the Trial group, a Biorepair Total Protective toothpaste containing zinc carbonate-substituted hydroxyapatite crystals was used to test the efficacy of hydroxyapatite. In the Control group, a Curasept Biosmalto Caries & Erosion toothpaste containing fluoro-hydroxyapatite and magnesium strontium carbonate hydroxyapatite conjugated with chitosan was used to evaluate possible differences in the study outcomes based on their formulations [13,25]. The null hypothesis was accepted, as no significant intergroup differences were found for all the study variables. However, evaluating the DP score, a significant reduction in enamel demineralization was found in the Trial group after 1 month of treatment, while in the Control group, no differences were found. This result suggests that the Trial toothpaste could probably lower caries incidence with respect to the Control toothpaste in the short-term application. This result is particularly relevant as the DIAGNOdent Pen provides a quantitative assessment of tooth mineralisation and health [33]. The Control toothpaste, even though a gradual decrease was found, did not present a significant improvement, but exerted a sort of remineralising activity. These findings are associated with patient compliance but not strictly correlated with correct oral hygiene maneuvers, since brushing, even if not performed correctly, somehow manages to lead to the deposition of toothpaste on occlusal surfaces. Additionally, implementing hydroxyapatite for daily home use could be a valid option for the reduction in dental caries risk [34].

The PI also showed a statistically significant reduction in both groups. In the Trial group, the reduction was in the time frame T0-T2, while in the Control group, it occurred in T0-T1 comparison. This finding is interesting, but it should be evaluated with caution as it depends on the oral hygiene maneuvers and motivation of the patients. It can be affected by nonsurgical periodontal debridement, but even after 3 and 6 months from baseline, the results indicate that a correct maintenance of patients was achieved. The recording of periodontal indexes in the present study served to evaluate patients’ compliance with oral hygiene maneuvers, implicitly implying the use of toothpastes under evaluation.

Conversely, BoP, SAI, and BEWE showed no significant changes in either group. Considering the BoP, a considerable variability was found. However, the BEWE and SAI scores did not change and remained at acceptable levels [13].

The DC score, though which the ICDAS index was recorded, showed no significant changes throughout this study. The ICDAS index is specifically designed to detect advanced carious lesions, which may explain the lack of improvement observed in this relatively short study period [35,36]. The index requires careful clinical observation over time and its effectiveness as a measure of short-term remineralisation may be limited. Therefore, the inability of the present study to detect rapid changes in caries status using ICDAS [29,37] highlights the need for longer-term studies to fully understand the potential benefits of remineralising agents.

Previous research supports the idea that diagnostic tools such as DIAGNOdent provide more accurate information than traditional methods. For example, a study by Lee et al. [38] demonstrated that laser technology can detect early changes in dental mineralization, highlighting the advantage of objective measurements in caries diagnosis and treatment radiographs. However, in the present study, it was used for the evaluation of fissures and pits of permanent first molars; therefore, further evaluations are required by analyzing more extended lesions. Other studies have examined variables such as duration of use and patient compliance, which may influence treatment duration and clinical outcomes [37]. A better understanding of these factors would provide a broader and more detailed perspective on the efficacy of remineralising agents and help to refine preventive and therapeutic strategies. In general, pediatric care can benefit from the home use of hydroxyapatite-based toothpastes; therefore, the current literature strongly recommends their use [34]. The fact that there is no adverse effect due to fluoride intake makes it suitable for children [13]. However, it should also be taken into account that when it is not possible to remineralise enamel lesions, the resin infiltration technique is the most suitable for the resolution, given its stability over time [39].

The limitations of this study include the sample that includes also pediatric patients, whose compliance and adherence to the home protocol should be accurately assessed.

The age range of the participants is quite wide, from 6 to 18 years, and this implies that brushing techniques and eating habits may potentially differ by age.

Still, the use of a toothpaste could not be sufficient to release an adequate amount of hydroxyapatite from both groups, and oral pH together with saliva composition could alter the deposition of the active ingredient. Furthermore, longer follow-ups and additional outcomes are desirable to assess the efficacy of caries prevention [40].

Therefore, future studies should consider delivering the experimental toothpaste in addition to a prolonged treatment that allows a correct deposition of the active ingredient. Moreover, the evaluation of remineralising treatments also after preventive procedures, such as pits and fissures sealants, could be considered [41,42].

## 5. Conclusions

The tested remineralising treatments were effective in reducing the degree of demineralization assessed with DIAGNOdent Pen. According to the results of this study, the zinc carbonate hydroxyapatite-based treatment is suggested in the home protocol for pediatric patients’ oral hygiene as it showed a faster action in the short-term period (T0–T1). No significant changes were found for DIAGNOcam evaluation and for the oral indexes, with the exception of the Plaque Index.

## Figures and Tables

**Table 1:** Products tested in this study and their compositions.

Toothpaste	Manufacturer	Composition
Biorepair Total Protective Repair	Coswell S.p.A., Funo di Argelato, BO, Italy	Zinc hydroxyapatite, aqua, aroma, glycerin, sorbitol, hydrated silica, silica, cellulose gum, tetrapotassium pyrophosphate, sodium methyl cocoyl taurate, sodium myristoyl sarcosinate, sodium saccharin, citric acid, phenoxyethanol, benzyl alcohol, sodium benzoate.
Curasept Biosmalto Caries Abrasion & Erosion	Curasept S.p.A, Saronno, VA, Italy	Purified water, glycerin, xylitol, fluoro hydroxyapatite, magnesium-strontium-carbonate hydroxyapatite conjugated with chitosan, cellulose gum, cocamidopropyl betaine, potassium acesulfame, xanthan gum, aroma, phenoxyethanol, sodium benzoate, aroma, citric acid.

**Table 2:** Descriptive and inferential statistics of the dental parameters recorded with DIAGNOdent Pen and DIAGNOcam in this study.

Group	Time	DP Mean (SD)	DC Mean (SD)
Trial	T0	17.06 (10.25) ^a^	0.64 (0.77) ^a,b^
	T1	10.39 (7.63) ^b,c,d^	0.63 (0.75) ^a,b^
	T2	8.39 (6.58) ^c,d^	0.48 (0.62) ^a,b^
	T3	8.19 (4.77) ^c,d^	0.38 (0.56) ^a^
Control	T0	12.46 (6.93) ^a,b^	0.78 (0.83) ^b^
	T1	10.79 (6.21) ^b,c^	0.78 (0.83) ^b^
	T2	9.53 (5.41) ^b,c,d^	0.64 (0.75) ^a,b^
	T3	8.98 (5.05) ^b,c,d^	0.56 (0.65) ^a,b^

Means with same lowercase superscript letters do not show statistical intergroup and intragroup significances. Abbreviations: DP, DIAGNOdent Pen score; DC, DIAGNOcam score.

**Table 3:** Descriptive and inferential statistics of the visual parameters recorded in this study.

Group	Time	SAI Mean (SD)	PI Mean (SD)	BoP Mean (SD)	BEWE Mean (SD)
Trial	T0	0.21 (0.69) ^a^	70.4 (29.67) ^a^	7.05 (17.14) ^a^	0.74 (0.52) ^a^
	T1	0.18 (0.57) ^a^	41.65 (18.92) ^a,b,c^	0.50 (2.24) ^a^	0.63 (0.54) ^a^
	T2	0.14 (0.44) ^a^	37.6 (16.62) ^b,c,d^	0.00 (0.00) ^a^	0.48 (0.53) ^a^
	T3	0.11 (0.39) ^a^	39.45 (21.79) ^c,d^	1.10 (3.08) ^a^	0.45 (0.53) ^a^
Control	T0	0.11 (0.45) ^a^	67.5 (31.53) ^a,c^	6.45 (11.56) ^a^	0.59 (00.59) ^a^
	T1	0.11 (0.45) ^a^	34.5 (13.93) ^b,d^	0.60 (02.26) ^a^	0.56 (0.59) ^a^
	T2	0.08 (0.31) ^a^	37.4 (15.19) ^b,c,d^	1.00 (3.08) ^a^	0.53 (0.55) ^a^
	T3	0.06 (0.29) ^a^	39.5 (20.63) ^b,c,d^	3.15 (8.31) ^a^	0.50 (0.55) ^a^

Means with same lowercase superscript letters do not show statistical intergroup and intragroup significance. Abbreviations: SAI, Schiff Air Index; PI, Plaque Index; BoP, bleeding on probing; BEWE, basic erosive wear examination.

## Data Availability

Data are available upon reasonable request to the corresponding authors.
